# Screening for subjective cognitive decline in the elderly via subjective cognitive complaints and informant-reported questionnaires: a systematic review

**DOI:** 10.1186/s12871-021-01493-5

**Published:** 2021-11-10

**Authors:** Sara Wasef, Isabelle Laksono, Paras Kapoor, David Tang-Wei, David Gold, Aparna Saripella, Sheila Riazi, Sazzadul Islam, Marina Englesakis, Jean Wong, Frances Chung

**Affiliations:** 1 Department of Anesthesia and Pain Management, Toronto Western Hospital, University Health Network, University of Toronto, Toronto, ON Canada; 2 Department of Neurology, University Health Network, University of Toronto, Toronto, ON Canada; 3 Krembil Neuroscience Centre, Toronto Western Hospital, University Health Network, University of Toronto, Toronto, ON Canada; 4 Library & Information Services, University Health Network, Toronto, ON Canada; 5 Department of Anesthesia and Pain Management, Women’s College Hospital, University of Toronto, Toronto, ON Canada

**Keywords:** Mild cognitive impairment, Elderly, Subjective cognitive complaints, Screening, Subjective cognitive decline, Reoperative screening

## Abstract

**Background:**

Subjective cognitive decline may represent at-risk persons progressing to mild cognitive impairment (MCI), which can be exacerbated by effects of anesthesia and surgery. The objective of this systematic review is to identify the most common questions in subjective cognitive complaint and informant-reported questionnaires used in assessing cognitive impairment of elderly patients that are correlated with standardized tests for cognitive impairment screening.

**Methods:**

We searched Medline, PubMed, Embase, Cochrane Central Register of Controlled Trials, Cochrane Database, Emcare Nursing, Web of Science, Scopus, CINAHL, ClinicalTrials.Gov, and ICTRP between September 20, 2005 to August 31, 2020. We included studies that evaluated subjective cognitive complaints and informant-reported questions in elderly patients.

**Results and conclusion:**

A total of 28,407 patients were included from 22 studies that assessed 21 subjective complaint questionnaires and nine informant-reported questionnaires. The most common subjective cognitive complaints were those assessing anterograde memory, closely followed by perceptual-motor function and executive function. The most common informant-reported questions were those assessing executive function, temporal orientation, and anterograde memory. Questions assessing learning and memory were most associated with results from standardized tests assessing cognitive impairment. Assessing learning and memory plays a key role in evaluating subjective cognitive decline in elderly patients. Delivering subjective cognitive complaints questions to elderly patient preoperatively may aid in screening for those exhibiting cognitive signs, and in turn are at risk of postoperative complications. Thus, the results from this review contribute to knowledge for healthcare professionals regarding the use of subjective cognitive complaints and informant-reported complaints in preoperative settings.

**Supplementary Information:**

The online version contains supplementary material available at 10.1186/s12871-021-01493-5.

## Background

Subjective cognitive decline in older persons, is described as the stage before the subsequent mild cognitive impairment (MCI) stage where apparently healthy persons report subjective cognitive complaints in the absence of objective evidence of cognitive impairment [1–8]. Its clinical presentation includes subjective self-reported impairment and decreased performance on objective cognitive screening tools such as the Mini-Mental State Examination (MMSE), Montreal Cognitive Assessment (MoCA), and Mini-Cog [9–12].

The elderly are particularly vulnerable to perioperative complications which get exacerbated by the physiological stress brought upon by surgery and anesthetics, with the risk of cognitive impairment being even higher after hospitalization [13–15]. Screening for the earliest identifiable cognitive changes among elderly patients is considered important to identify those who require a formal neurocognitive assessment and early management [16]. Cognitive decline is substantially underdiagnosed since objective screening tools can be challenging to administer in busy preoperative settings [17]. Thus, screening for subjective self-reported impairment can be advantageous due to easy administration and low cost [8]. Screening for subjective cognitive decline can be of significant value to identify at-risk patients for MCI since it may be one of the earliest demonstrations of Alzheimer’s disease [18–20]. Thus, a concise set of questions that serve to screen patients for potential cognitive impairment prior to surgery can determine whether further neuropsychological assessment is required. This knowledge may lead to a better understanding of postoperative complications and optimization of preoperative management influencing surgical and anesthetic approaches [15, 21, 22].

The objective of this systematic review is to identify the most common subjective questions in subjective cognitive complaints that are correlated with standardized tests. These results will assist in determining a couple of possible questions best correlated with subjective cognitive decline that we can utilize in the preoperative assessment if we suspect the elderly surgical patients may have MCI.

## Main text

This study was performed in accordance with the Preferred Reporting Items for Systematic Reviews and Meta-Analyses (PRISMA) guidelines [23]. The protocol of this systematic review was registered in the International Prospective Register of Systematic Reviews (PROSPERO) (CRD42020205893).

### Study selection criteria

The inclusion criteria were: 1) randomized and non-randomized controlled studies, observational studies; 2) patients aged 50 years old or more; and 3) studies with subjective cognitive complaints and/or informant-reported questions in the form of a questionnaire/set of questions. Articles not written in English and case reports or series were excluded.

### Search strategy

**Fig. 1:**
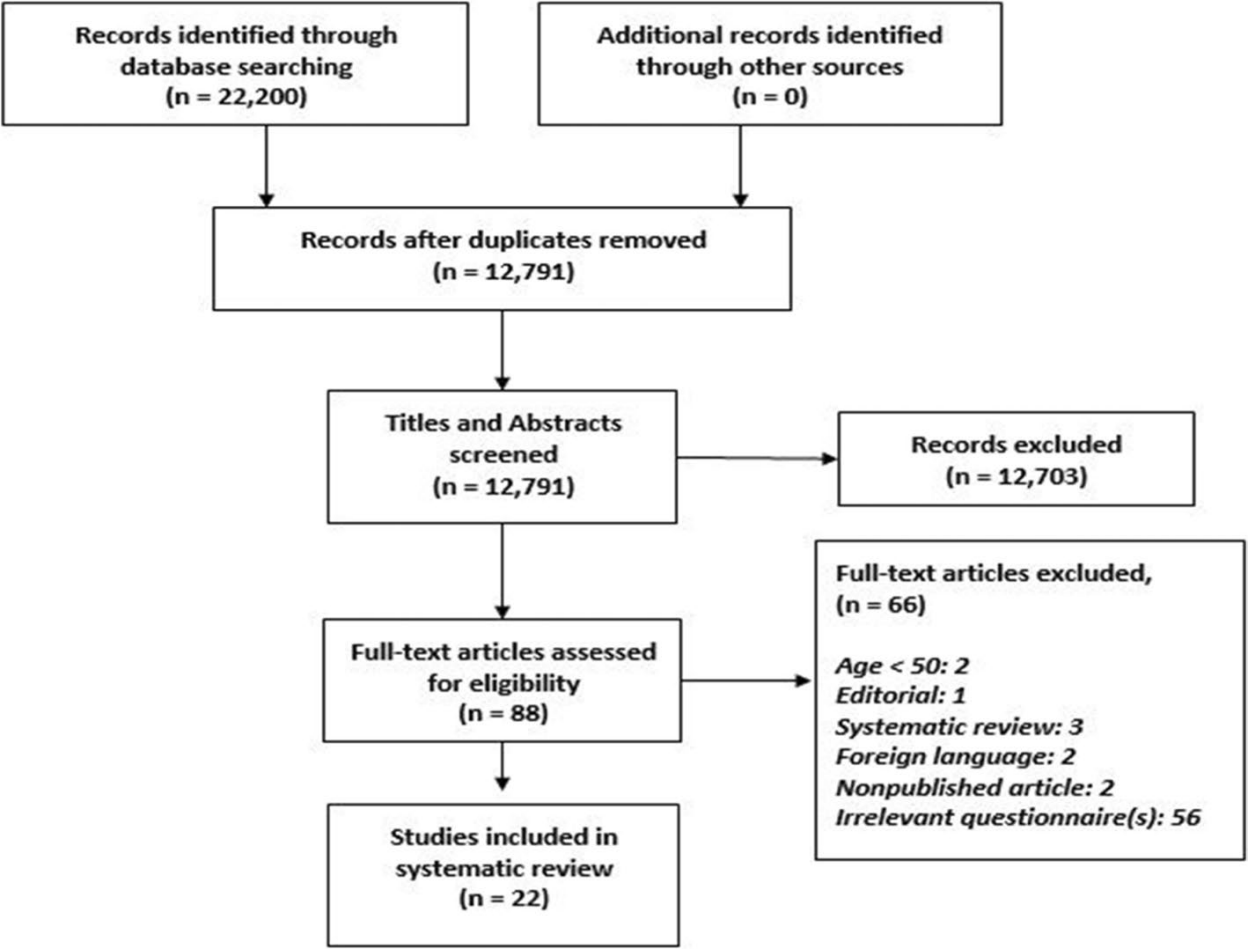
PRISMA Diagram

We searched Medline, Pubmed, Embase, Cochrane Central Register of Controlled Trials, Cochrane Database of Systematic Reviews, Emcare Nursing, Web of Science, Scopus, CINAHL (The Cumulative Index to Nursing and Allied Health Literature), ClinicalTrials.Gov, and ICTRP (international Clinical Trials Registry Platform) for published and unpublished studies. The search strategy was developed with the help of an information specialist who is experienced in search strategy development (ME). The searching process followed the Preferred Reporting Items for Systematic Reviews and Meta-analyses (PRISMA) guideline (Fig. 1) [23]. We used both MeSH and free text terms to identify relevant articles. Preliminary searches were conducted, and full-text literature was examined for potential keywords and controlled vocabulary terms using Medical Subject Headings for Medline (MeSH) and EMTREE vocabulary words for Embase. Database searches were restricted from September 20, 2005 to January 23, 2020. Updated search was done up to August 31st, 2020. The search strategy used controlled vocabulary terms and text word terms for each of the research topic components, ‘(Cognition OR Cognitive Dysfunction) AND (Questionnaires OR Surveys) AND (Patients or Alternates Complaints) AND (Aged or Elderly)’. Detailed search strategy is provided in the supplementary (S-1).

### Study process

The study authors prepared the pilot tested data collection form with the standard instruction for screening of the title, abstract, full text, data collection, and data analysis. Two reviewers (AS, SW) did title, abstract, and full text screening independently using Endnote and Rayyan. Arising conflicts were resolved by the senior author (FC).

### Data extraction

Data was extracted using standardized data collection form. It was conducted by two authors (SW and IL), and reviewed by the senior author (FC). Study characteristics such as author, publication year, country of origin, study design, total sample size, study setting, inclusion criteria, and conditions being investigated were collected. The conditions being investigated were defined according to the DSM-5 [24]. Dementia is renamed as “major neurocognitive disorder” and MCI as “mild neurocognitive disorder”. MCI is defined as cognitive decline in one or more cognitive domains that does not interfere with independence in daily activities or meet criteria for dementia. Patient characteristics such as age and gender were extracted. Information about the questionnaires such as title, number of questions, questionnaire items, validation, association, prevalence of subjective cognitive complaints (SCCs) and answers to informant-reported questions were extracted.

Data on validation and prevalence were extracted for the subjective cognitive complaints questionnaire as a whole as well as for each individual question. Moreover, questions of each questionnaire were extracted. Questions are grouped under the six domains of cognitive function described by the Neurocognitive Work Group which include: 1) complex attention, 2) executive function, 3) learning and memory, 4) language, 5) perceptual–motor function, and 6) social cognition. Complex attention includes skills such as the ability to maintain attention and information processing speed. Executive function includes skills such as planning and decision-making. Learning and memory refers to recall and implicit learning tasks. Perceptual-motor function includes visual perception, such as spatial orientation. Language includes object naming such as selecting the right words to describe something [25].

### Search results

A complete search of the articles is summarized in the PRISMA flow chart (Fig. 1). The electronic search strategy in the selected databases yielded 22,200 articles. Deduplication was conducted resulting in 12,791 remaining articles. Our search has focused on studies which included questionnaires evaluating subjective cognitive complaints and/or informant-reported complaints as well as analysis of commonality of specific complaints/questions and/or links between the questions/complaints and actual cognitive impairment. Title and abstract screening were conducted resulting in 312 abstracts screened with 88 articles remaining. Full-length screening of the 88 articles was conducted, with 22 articles being included for the review. Reasons for exclusion are listed in Fig. 1.

### Patient and study characteristics

This review included 22 studies based on our search. Among the 22 studies, 20 were prospective and two were retrospective trials with a total of 28,407 patients (Table 1). The majority of studies were from the United States (*n* = 7), with the remainder from Spain (*n* = 3), South Korea (*n* = 3), Hong Kong (*n* = 1), Greece (*n* = 1), Singapore (*n* = 1), China (*n* = 1), South Africa (*n* = 1), Italy (*n* = 1), Czech Republic (*n* = 1), France (*n* = 1) and Norway (*n* = 1) [26–47]. The mean age of patients was 75.0 (8.3) years with 84% female, largely due to a female only study including 16,964 subjects [41]. Nine studies recruited participants from home and/or the community, four recruited from hospitals and clinics, while five recruited from both clinics and community (Table 1). Eight studies investigated dementia, two investigated Alzheimer’s Disease, 13 investigated MCI (defined as general cognitive decline), and five investigated amnestic MCI (Table 2). Of the 22 studies, 14 investigated subjective cognitive complaints, [26–28, 30–32, 34, 38, 39, 41, 44–47] four examined both subjective cognitive complaints as well as informant-reported questions, [29, 33, 36, 43] and four explored informant-reported questions [35, 37, 40, 42]. Among these 22 studies, 21 questionnaires on subjective cognitive complaints and nine questionnaires on informant reports were assessed (Table 2). All questionnaires included at least one subjective memory complaint item, however, only five subjective cognitive complaints and one informant-report questionnaires included subjective memory items exclusively [26, 27, 32, 38, 42]. The questionnaires were compared to the result of known cognitive measures. In 14 of the studies, the cognitive measure was a neuropsychological battery of tests whose test content differed based on the study. The questionnaires ranged from 1 to 28 questions. The complete list of questions is reported in Table S-2, while the characteristics of the subjective cognitive complaint questionnaires are reported in Table S-3.

**Table 1:** Demographics and study characteristics

Study (author, year, location)	Study type	Sample size (n)	Age, years Mean ± SD	Gender (% female)	Setting	Outcome
Bosnes, 2020 (Norway) [26]	PC	106	73.4 ± 8.4	53	NR	Dementia
Hess, 2020 (US) [27]	PC	124	73.59 ± 6.26	82	Community Outpatient clinic	Dementia
Guerdoux-Ninot, 2019 (France) [28]	PC	488	Healthy controls: 56.5 ± 15.1 Functional patients: 57.1 ± 11.5 naMCI patients: 69 ± 8.5 aMCI patients: 70.6 ± 10.5 AD patients: 72.8 ± 7.4	Healthy controls: 106 Functional patients: 66 na-MCI patients: 42 a-MCI patients: 48 AD patients: 32	Community Outpatient clinic	aMCI naMCI AD
Kim, 2019 (South Korea) [29]	PC	420	75 ± 6	46	Outpatient clinic	Dementia MCI
Howland, 2017 (US) [30]	PC	281	78	72	Outpatient clinic Community	MCI
Markova, 2017 (Czech Republic) [31]	RC	340	75 ± 8	55	Community	MCI
Papaliagkas, 2017 (Greece) [32]	PC	81	Older adults: 70 ± 4 Older-old adults: 84 ± 3	Older adults: 42 Older-old adults: 64	NR	MCI
Yim, 2017 (South Korea) [33]	PC	814	Cognition intact: 69 ± 7. MCI: 73 ± 7. Dementia: 73 ± 9 Overall cognitive disorder: 73 ± 9	64	Outpatient clinic	Dementia MCI
Avila-Villanueva, 2016 (Spain) [34]	PC	844	Control: 74 ± 4 MCI: 76 ± 4	Control: 63 MCI: 50	Community	MCI
Tew, 2015 (Singapore) [35]	PC	245	Dementia: 77 ± 8 No dementia: 68 ± 7	Dementia: 64 No dementia: 72	Outpatient clinic Community	Dementia
Valech, 2015 (Spain) [36]	PC	217	Control: 65 ± 8 Pre-AD: 69 ± 8 NonA-CI: 63 ± 10 A-CI: 70 ± 8	Control: 68 Pre-AD: 79 NonAB-CI: 42 AB-CI: 62	Outpatient clinic Community	aMCI
Li, 2013 (China) [37]	PC	356	72 ± 9	58	Community	MCI
Ramlall, 2013 (South Africa) [38]	PC	140	75 ± 9	69	Community	Dementia MCI
Snitz, 2012 (US) [39]	PC; secondary analysis	3495	78 ± 7	62	Community	aMCI
Abbate, 2011 (Italy) [40]	RC	119	77 ± 6	62	Outpatient clinic	MCI
Amariglio, 2011 (US) [41]	PC	16,964	74	100	Community	MCI
Ayalon, 2011 (US) [42]	PC	647	Cognition intact: 77 ± 0 CIND: 81 ± 1 Dementia: 84 ± 1	66	NR	Dementia MCI
Gavett, 2011 (US) [43]	PC	384	Initial visit: 70 ± 7 Final visit: 73 ± 7	100	Community	MCI
Calabria, 2010 (Spain) [44]	PC	112	71 ± 6	78	Community	aMCI
Youn, 2009 (S. Korea) [45]	PC	1651	74 ± 8	57	NR	Dementia
Snitz, 2008 (US) [47]	PC	276	73 ± 6	58	Outpatient clinic	aMCI
Lam, 2005 (Hong Kong) [46]	PC	306	79 ± 7	NR	Community	AD MCI

*Abbreviations*: *A-CI* Amyloid cognitive impairment, *AD* Alzheimer’s Disease, *aMCI* amnestic mild cognitive impairment, *CIND* cognitive impairment not dementia, *MCI* mild cognitive impairment, *naMCI* non-amnestic mild cognitive impairment, *NonAB-CI* Non-amyloid impairment, *NR* Not Reported, *PC* Prospective cohort, *Pre-AD* preclinical Alzheimer’s disease, *RC* Retrospective cohort, *SD* standard deviation

**Table 2:** Most common questions

Neurocognitive Domain	Question Category	Number of Studies (subjective cognitive complaints)	Number of Studies (informant-report questions)
Learning and Memory	Anterograde memory (e.g. do you/does the patient have difficulty remembering things that have happened recently?)	11	4
	Ability to remember and/or keep appointments (e.g. do you/does the patient have trouble remembering appointments?)	9	2
	Forgetfulness of common objects (e.g. do you/does the patient lose objects more often than you did previously?)	7	0
	Temporal orientation (e.g. do you/does the patient have trouble remembering the time/date?)	5	5
	Comparing own memory to others of similar age (e.g. do you/does the patient think that your memory is poorer than that of other people your age?)	4	0
	Remembering routine tasks (e.g. do you/does the patient have trouble remembering how to turn off the stove or lights?)	4	0
Perceptual-Motor Function	Spatial orientation (e.g. do you/does the patient have trouble finding your way around familiar streets?)	9	1
Executive Function	Executive function (e.g. do you/does the patient have trouble working household appliances?)	7	6
Language	Language (e.g. do you/does the patient have trouble finding the right word to describe something you know well?)	6	2
Complex Attention	Ability to follow a conversation (e.g. do you/does the patient have trouble following TV program or a book?)	6	3

### Prevalence of subjective cognitive complaints/informant-reported questions and validity of questionnaires

Among the five subjective cognitive complaints questionnaires that reported prevalence of individual complaints, the items that demonstrated a prevalence rate over 30% among study participants are: (1) remembering names of people you met only recently; (2) how well you remember things compared to a year ago; (3) considering memory to be worse than others of a similar age; (4) finding the right word to use to describe something you know well; and (5) forgetting where things are placed; inability to recall the names of good friends; and difficulties with recalling past events (Table 2) [31, 39, 41, 46, 47].

Regarding questionnaire validity, various cognitive measure assessment tools used to validate questionnaires are listed in Table S-3. Fifteen studies reported validation data for their respective questionnaires against cognitive measures [26, 28, 29, 31–33, 35–38, 45–47]. Eight of these included validated questionnaires which evaluated subjective cognitive complaints only. One study reported prevalence of individual informant-reported questions. The items that had a prevalence rate over 30% are: (1) remembering events that happened a short time ago; (2) remembering things that happened in the past; (3) being able to pay attention and concentrate; and (4) being able to remember whether mistakes were made in performing specific tasks or household chores (Table 2) [40]. Regarding the validity of questionnaires, six out of the eight studies which included informant-reported questionnaires reported data on validity of the questionnaires [33, 35–37, 40, 42]. Due to the heterogeneity in validation data reporting, conducting a meta-analysis on validity of the different questionnaires assessing subjective cognitive complaints/informed-reported questions was not feasible.

### Most frequent questions utilized in subjective cognitive complaints and informant-reported questions

Overall, ten types of questions were found to be more frequently utilized in these questionnaires. These questions were grouped under the six domains of cognitive function described by the Neurocognitive Work Group: 1) complex attention, 2) executive function, 3) learning and memory, 4) language, 5) perceptual–motor function, and 6) social cognition (Table 2) [25]. Two reviewers (IL and SW) independently classified items by cognitive domains and inter-rater reliability was 100%. The most common category was learning and memory, followed by perceptual-motor function and executive function. Specifically, anterograde memory was the most common question type among all sub-categories. Both anterograde memory and temporal orientation were grouped under learning and memory. Anterograde memory refers to the patients’ ability to remember events that recently took place. Temporal orientation refers to the patients’ awareness of date and time. Spatial orientation was grouped under perceptual-motor function and refers to the patient’s ability to navigate familiar surroundings. Ability to work familiar appliances was grouped under executive function, while ability to remember appointments was also grouped under learning and memory. In studies that reported on informant-reported complaints, concerns with executive function, temporal orientation, and anterograde memory were the most common.

### Positive associations in different domains of cognitive questions

Of the 15 articles which reported on anterograde memory, seven showed a positive association between the subjective/informant-reported questions related to anterograde memory and cognitive measure tests (Table S-3) [31, 34, 38, 40, 41, 45, 47]. Three studies reported positive associations between questions evaluating executive function and cognitive measures, with another three studies reporting on language [34, 38–41]. A positive association was reported in four studies between spatial orientation and cognitive measures, with forgetfulness of common objects and comparison of memory to those of similar age being reported in two studies, and one study reporting on [31, 40, 41, 44, 45] ability to remember/keep appointments and remembering routine tasks. Studies with validation data are indicated in Table S-3.

## Discussion

We evaluated the use of subjective cognitive decline in screening for cognitive decline in 22 studies with 21 different subjective complaint questionnaires and nine informant-reported questionnaires. Among subjective cognitive complaints questionnaires, the most frequently assessed neurocognitive domain was learning and memory, followed by perceptual-motor function. Among informant-reported questionnaires, the most frequently assessed neurocognitive domain was learning and memory, followed by executive function. Among questionnaires with individual subjective cognitive complaints items, questions assessing anterograde memory were most positively associated with overall cognitive status.

Three studies reported the prevalence of at least one subjective cognitive complaints item. The prevalence of subjective cognitive complaints in elderly healthy volunteers from community settings are over 70% in two studies, whereas one study reported 35.9% [31, 41, 47]. This may be due to the use of seven or more items in these studies with a high prevalence versus a single-item questionnaire. The high prevalence of subjective cognitive complaints in this patient population emphasizes the importance of characterizing subjective complaints and assessing their utility in predicting overall cognitive status preoperatively.

The Neurocognitive Work Group’s six cognitive domains were used to group subjective cognitive decline questions [25]. The learning and memory domain is the most well-known as it is associated with the frequent amnestic presentation of Alzheimer’s disease [6]. An example question assessing this domain is “are you having trouble remembering things that happened a few days ago?”. Perceptual-motor function can be assessed with questions such as “are you able to navigate around familiar streets?”, and an example of a question assessing executive function is, “are you able to plan large family events successfully?”. Our findings of the most common questions are consistent with studies reporting that subjective memory complaints make up the majority of all SCCs [8].

Seven studies described positive associations between anterograde memory in subjective cognitive complaints and overall cognitive decline in patients [34, 38, 40, 41, 44, 45, 47]. One study identified a positive association between anterograde memory in informant-reported complaints and cognitive decline [40]. This is consistent with the fact that Alzheimer’s disease and amnestic MCI are defined by deficits in memory, and that these conditions make up the majority of dementia and MCI cases among elderly patients [48, 49]. The four studies that looked at both subjective cognitive complaints and informant-reported questions compared questionnaires as a whole. Three of the four studies found that informant questionnaires outperformed patient-reported questionnaires [29, 36, 43]. Yim et al. suggested that a combined questionnaire was found to have better screening accuracy compared to each questionnaire individually [33]. These findings are consistent with previous suggestions that informant-reports may provide a more robust version of the patient’s cognitive status due to lack of bias [45, 50].

Interestingly, Snitz et al. completed a study evaluating 14 subjective cognitive complaints with the majority related to memory, and another one evaluating 27 common subjective cognitive complaints items which includes multiple neurocognitive domains in addition to memory complaints [39, 47]. The 27 items predicted cognitive dysfunction more accurately, suggesting that assessing memory decline alone is not as effective as assessing multiple neurocognitive domains. Although memory-related questions appear to be the most predictive, other questions that include other domains likely need to be included in order to screen for non-amnestic MCI and non-Alzheimer’s disease dementias [51]. For feasibility, the time of completion of the screening questionnaire needs to be short. None of those included studies reported questionnaire administration time.

Physicians are often unaware of cognitive impairment, in 40% of their cognitively impaired patients [51–53]. Early screening in all clinical populations allows for earlier intervention that is associated with a delay in disease progression [16]. Anesthesiology and surgery are associated with increasing rate of cognitive decline, as they have demonstrated that they induce an age-dependent neuroinflammatory response [54–56]. As elderly individuals are undergoing surgeries at a progressively increased rate and some may have unrecognized pre-existing cognitive impairment, it is important to screen for the earliest identifiable cognitive changes in those considering surgery [12, 52]. Early tests of subjective cognitive decline can be used to warrant further evaluation. Thus, in elderly patients with suspected cognitive decline, delivering questions which assess for subjective cognitive complaints may aid in screening of those with potential cognitive decline who may experience a higher rate of morbidity postoperatively. Memory type subjective cognitive complaints are important to use along with assessment questions from other domains as well.

The main drawback of using subjective cognitive complaints to screen for cognitive decline is that this patient population often lacks insight into their difficulties, which may yield inaccurate responses, emphasizing the role of informant-reported complaints [8]. Further work is needed to determine which individual SCC questions are most sensitive in screening for cognitive decline in this patient population preoperatively. Nevertheless, our work found that some of the most important questions are those which evaluate anterograde memory, spatial and temporal orientation, as well as those assessing executive functioning. A proposed questionnaire for future validation studies could include questions which highlight the aforementioned domains. For example, a future short questionnaire can include the following questions: “Do you have difficulty remembering things that have happened recently?”; “Do you have trouble remembering the time/date?”; and “Do you have trouble working household appliances?”

### Limitations

This systematic review has some limitations. In these 22 studies, different subjective cognitive complaints questionnaires were utilized, making it difficult to compare results. Second, the studies were heterogeneous with various clinical settings with variability in types of MCI. Third, the majority of studies validated entire questionnaires as a whole instead of assessing individual questions, making it difficult to identify the more valid questions.

## Conclusions

Our review demonstrates that the most frequently assessed and positively correlated domain by subjective cognitive complaints question was learning and memory. We found that questions used to assess subjective complaints should include a few of the most sensitive subjective cognitive complaints questions, especially those assessing memory, such as, “are you having trouble remembering things that happened a few days ago?”. Our results contribute to knowledge for healthcare professionals regarding the use of subjective cognitive complaints and informant-reported complaints to assess for subjective cognitive decline in elderly patients in preoperative settings.

## Supplementary Information

**Additional file 1: S1**: Search Strategy.

**Additional file 2: Table S-2**: List of Subjective Cognitive Complaint Questions.

**Additional file 3: Table S-3**: Characteristics of Subjective Cognitive Complaint Questionnaire. *Abbreviations:* AD8: Alzheimer Disease 8; A-MIC, abbreviated memory inventory for the Chinese; CFQ, Cognitive Failures Questionnaire; IADLs - Instrumental Activities of Daily Living; IQCODE, Informant Questionnaire on Cognitive Decline in the Elderly; KDSQ-C, Korean Dementia Screening Questionnaire-Cognition; NR, Not Reported; PRMQ, Prospective and Retrospective Memory Questionnaire; PROMIS, Patient-Reported Outcomes Measurement Information System; SCC, subjective cognitive complaint; subjective cognitive decline, subjective cognitive decline; SIRQD, Seoul Informant Report Questionnaire for Dementia; SMA, subjective memory assessment; SMC, subjective memory complaint; SMCQ, Subjective Memory Complaints Questionnaire; MMQ, Meta-Memory Questionnaire.

## Data Availability

All data generated or analysed during this study are included in this published article and supplementary files.
