# Supplementary material for: Screening for subjective cognitive decline in the elderly via subjective cognitive complaints and informant-reported questionnaires: a systematic review
Source: BMC Anesthesiol. 2021 Nov 10;21:277. doi: 10.1186/s12871-021-01493-5 (PMC8579566; doi:10.1186/s12871-021-01493-5)
Supplement: Supplementary file 1 — Additional file 1: S1: Search Strategy. [file 12871_2021_1493_MOESM1_ESM.docx]

**S1 – Search Strategy for “Screening for Subjective Cognitive Decline in the Elderly via Subjective Cognitive Complaints and Informant-Reported Questionnaires: A Systematic Review”**

Medline Search Strategy for: **Subjective cognitive complaints and elderly**; limited to English, human, years=2000- present

**Original Searches conducted April 14, 2020**

| Databases | **Database Dates covered** | **Date Database  was searched** | **# Citations** |
| --- | --- | --- | --- |
| Medline (OvidSP) | 1946 – April 13, 2020 | April 14, 2020 | 4380 |
| ePub Ahead of Print / Medline In-Process & Other Non-Indexed Citations (OvidSP) | 2020 April 13 | April 14, 2020 | 404 |
| Embase (OvidSP) | 1947 – April 13, 2020 | April 14, 2020 | 6832 |
| Ovid EmCare Nursing (Ovid) | 1995 – Present | April 14, 2020 | 5397 |
| Cochrane Central Register of Controlled Trials (OvidSP) | 1991 – Present | April 14, 2020 | 486 |
| Cochrane Database of Systematic Reviews (OvidSP) | 2005 – Present | April 14, 2020 | 2 |
| PsycINFO (Ovid) | 1806 – April Week #1 2020 | April 14, 2020 | 1716 |
| Web of Science (Clarivate) | 1900 – April 13, 2020 | April 14, 2020 | 758 |
| CINAHL (EbscoHost) | 1982 – Present | April 14, 2020 | 1204 |
|  |  | Totals: | 21179 |

**Update Searches conducted August 24, 2020**

| Databases | **Database Dates covered** | **Date Database  was searched** | **# Citations** |
| --- | --- | --- | --- |
| Medline (OvidSP) | 1946 – August21, 2020 | August 24, 2020 | 176 |
| ePub Ahead of Print / Medline In-Process & Other Non-Indexed Citations (OvidSP) | 2020 August 21 | August 24, 2020 | 83 |
| Embase (OvidSP) | 1947 – August 21, 2020 | August 24, 2020 | 393 |
| Ovid EmCare Nursing (Ovid) | 1995 – Present | August 24, 2020 | 184 |
| Cochrane Central Register of Controlled Trials (OvidSP) | 1991 – Present | August 24, 2020 | 9 |
| Cochrane Database of Systematic Reviews (OvidSP) | 2005 – Present | August 24, 2020 | 1 |
| PsycINFO (Ovid) | 1806 –August Week #3 2020 | August 24, 2020 | 52 |
| Web of Science (Clarivate) | 1900 – August 21, 2020 | August 24, 2020 | 78 |
| CINAHL (EbscoHost) | 1982 – Present | August 24, 2020 | 45 |
|  |  | Totals: | 1021 |

Combined search results:

| Search results | April 14, 2020 | Totals: | 21179 |
| --- | --- | --- | --- |
| Search results | August 24, 2020 | Totals: | 1021 |
|  |  | **Combined Total** | **22200** |

# Medline

Ovid MEDLINE(R) 1946 to August 21, 2020

| **#** | **Searches** |  | **Results** |
| --- | --- | --- | --- |
| 1 | cognition disorders/ or mild cognitive impairment/ | Terms Related to Cognition / Cognitive Disorders / Cognitive Dysfunction OR manifestations of Cognitive Impairment | 81241 |
| 2 | (cognitiv* adj2 impair*).mp,kw. |  | 59429 |
| 3 | (disorder* adj2 cognit*).mp,kw. |  | 68323 |
| 4 | (cognitiv* adj2 status).mp,kw. |  | 5259 |
| 5 | (cognitiv* adj2 dysfunc*).mp,kw. |  | 29195 |
| 6 | (cognitiv* adj2 function*).mp,kw. |  | 56777 |
| 7 | dyscognitiv*.mp,kw. |  | 48 |
| 8 | cognitive*.mp,kw. |  | 310194 |
| 9 | exp Cognition/ |  | 161203 |
| 10 | Cognitive Aging/ |  | 642 |
| 11 | cognition*.mp,kw. |  | 188613 |
| 12 | Confusion/ |  | 4790 |
| 13 | (confuse? or confusing or confusion*).mp,kw. |  | 51614 |
| 14 | Delirium/ |  | 9376 |
| 15 | delirium?.mp,kw. |  | 15802 |
| 16 | delirius.mp,kw. |  | 1 |
| 17 | delirious*.mp,kw. |  | 1212 |
| 18 | Hallucinations/ |  | 10850 |
| 19 | hallucinat*.mp,kw. |  | 16954 |
| 20 | exp Dementia/ |  | 166159 |
| 21 | dementia?.mp,kw. |  | 111209 |
| 22 | demented??.mp,kw. |  | 7924 |
| 23 | dementat*.mp,kw. |  | 5 |
| 24 | demenc???.mp,kw. |  | 1537 |
| 25 | (intellectual* adj2 declin*).mp,kw. |  | 299 |
| 26 | amentia???.mp,kw. |  | 87 |
| 27 | (mental* adj2 deteriorat*).mp,kw. |  | 1460 |
| 28 | (mental* adj2 acuit*).mp,kw. |  | 88 |
| 29 | (mental* adj2 impair*).mp,kw. |  | 2697 |
| 30 | (mental* adj2 complain*).mp,kw. |  | 247 |
| 31 | (memor* adj2 acuit*).mp,kw. |  | 15 |
| 32 | (memor* adj2 impair*).mp,kw. |  | 17179 |
| 33 | (memor* adj2 complain*).mp,kw. |  | 1398 |
| 34 | (memor* adj2 dysfunct*).mp,kw. |  | 1942 |
| 35 | (memor* adj2 disfunct*).mp,kw. |  | 4 |
| 36 | (mental* adj2 dysfunct*).mp,kw. |  | 449 |
| 37 | Memory/ |  | 67145 |
| 38 | exp Memory Disorders/ |  | 29582 |
| 39 | Mental Health/ |  | 38678 |
| 40 | (mental* adj2 health*).mp. |  | 166744 |
| 41 | Recognition, Psychology/ |  | 18350 |
| 42 | or/1-41 **[ Cognition / Cognition Disorders ]** |  | 856737 |
| 43 | patient reported outcome measures/ | Terms Related to surveys or questionnaires or other means of soliciting information from patients | 6178 |
| 44 | Self Report/ |  | 32826 |
| 45 | personal narrative/ |  | 5011 |
| 46 | interview/ |  | 28500 |
| 47 | Interview, Psychological/ |  | 15064 |
| 48 | Metacognition/ |  | 976 |
| 49 | exp Neuropsychological Tests/ |  | 177637 |
| 50 | exp Psychiatric Status Rating Scales/ |  | 84059 |
| 51 | Psychometrics/ |  | 75468 |
| 52 | data collection/ or focus groups/ or geriatric assessment/ or health impact assessment/ or interviews as topic/ or narration/ |  | 210086 |
| 53 | "Surveys and Questionnaires"/ |  | 466102 |
| 54 | self report*.mp. |  | 142931 |
| 55 | survey*.mp. |  | 921700 |
| 56 | questionnaire*.mp. |  | 668154 |
| 57 | question*4.mp. |  | 420035 |
| 58 | (patient* adj2 experienc*).mp. |  | 90314 |
| 59 | (inpatient* adj2 experienc*).mp. |  | 497 |
| 60 | (patient* adj2 perspectiv*).mp. |  | 10284 |
| 61 | (inpatient* adj2 perspectiv*).mp. |  | 54 |
| 62 | (patient* adj2 perception*).mp. |  | 12216 |
| 63 | (inpatient* adj2 perception*).mp. |  | 78 |
| 64 | (lived adj2 experienc*).mp. |  | 4943 |
| 65 | interview*.mp. |  | 341486 |
| 66 | report*3.mp. |  | 4573616 |
| 67 | screen*.mp,kw. |  | 716657 |
| 68 | or/43-67 **[ Questions / Surveys / Reports ]** |  | 6481627 |
| 69 | 42 and 68 [ Cognition + Questions ] |  | 385093 |
| 70 | patients/ and (alleg* or claim*3 or complain* or concern* or plaint* or protest* or subjective*).mp. | Terms related to patient or caregiver complaints | 1817 |
| 71 | inpatients/ and (alleg* or claim*3 or complain* or concern* or plaint* or protest* or subjective*).mp. |  | 1773 |
| 72 | (adult??? adj10 (alleg* or claim*3 or complain* or concern* or plaint* or protest* or subjective*)).mp. |  | 9173 |
| 73 | (patient??? adj10 (alleg* or claim*3 or complain* or concern* or plaint* or protest* or subjective*)).mp. |  | 127614 |
| 74 | (inpatient??? adj10 (alleg* or claim*3 or complain* or concern* or plaint* or protest* or subjective*)).mp. |  | 1880 |
| 75 | (guardian* adj10 (alleg* or claim*3 or complain* or concern* or plaint* or protest* or subjective*)).mp. |  | 152 |
| 76 | ((person or persons or person's or personal*) adj10 (alleg* or claim*3 or complain* or concern* or plaint* or protest* or subjective*)).mp. |  | 11121 |
| 77 | ((carer or carers or caregiver? or care giver?) adj10 (alleg* or claim*3 or complain* or concern* or plaint* or protest* or subjective*)).mp. |  | 2426 |
| 78 | (informant? adj10 (alleg* or claim*3 or complain* or concern* or plaint* or protest* or subjective*)).mp. |  | 327 |
| 79 | (respondent? adj10 (alleg* or claim*3 or complain* or concern* or plaint* or protest* or subjective*)).mp. |  | 2914 |
| 80 | (substitute adj2 decision* adj10 (alleg* or claim*3 or complain* or concern* or plaint* or protest* or subjective*)).mp. |  | 4 |
| 81 | (alternat* adj2 decision* adj10 (alleg* or claim*3 or complain* or concern* or plaint* or protest* or subjective*)).mp. |  | 7 |
| 82 | ((proxy or proxy's or proxies) adj10 (alleg* or claim*3 or complain* or concern* or plaint* or protest* or subjective*)).mp. |  | 292 |
| 83 | (aged adj10 (alleg* or claim*3 or complain* or concern* or plaint* or protest* or subjective*)).mp. |  | 3969 |
| 84 | (elderly adj10 (alleg* or claim*3 or complain* or concern* or plaint* or protest* or subjective*)).mp. |  | 3995 |
| 85 | (subjective* adj3 cognit* adj3 complain*).mp,kw. |  | 350 |
| 86 | (subjective* adj3 cognit* adj3 impair*).mp,kw. |  | 386 |
| 87 | (subjective* adj3 memory adj3 complain*).mp,kw. |  | 589 |
| 88 | (subjective* adj3 memory adj3 impair*).mp,kw. |  | 220 |
| 89 | (cognit* adj3 complain*).mp,kw. |  | 1422 |
| 90 | (memory adj3 complain*).mp,kw. |  | 1509 |
| 91 | or/70-90 **[ Patients or Alternates complaints about cognition or memory ]** |  | 158524 |
| 92 | 69 and 91 [ Cognition + Questions + Patients/Alternates ] |  | 11618 |
| 93 | exp aged/ or "aged, 80 and over"/ or frail elderly/ | Terms related to the elderly or aged or patients 65 years of age or older | 3127384 |
| 94 | Aging/ |  | 228949 |
| 95 | Cognitive Aging/ |  | 642 |
| 96 | exp Geriatrics/ |  | 30023 |
| 97 | exp Geriatric Assessment/ |  | 27703 |
| 98 | Geriatric Psychiatry/ |  | 2363 |
| 99 | exp Health Services for the Aged/ |  | 17721 |
| 100 | exp Geriatric Nursing/ |  | 13594 |
| 101 | "older than 1##".mp. |  | 98 |
| 102 | "older than 6#".mp. |  | 7274 |
| 103 | "older than 7#".mp. |  | 3285 |
| 104 | "older than 8#".mp. |  | 1239 |
| 105 | "older than 9#".mp. |  | 122 |
| 106 | ("over 1##" adj8 year?).mp. |  | 2340 |
| 107 | ("over 6#" adj8 year?).mp. |  | 10466 |
| 108 | ("over 7#" adj8 year?).mp. |  | 5014 |
| 109 | ("over 8#" adj8 year?).mp. |  | 2617 |
| 110 | ("over 9#" adj8 year?).mp. |  | 917 |
| 111 | ((old?? or advance?) adj (age or aging or ageing)).mp. |  | 75708 |
| 112 | ((old?? or elder?? or senior?) adj (patient? or citizen?? or person? or people or geriatric* or population?)).mp. |  | 188253 |
| 113 | (aged adj2 "10# years").mp. |  | 997 |
| 114 | (aged adj2 "6# years").mp. |  | 39125 |
| 115 | (aged adj2 "65 years").mp. |  | 17383 |
| 116 | (aged adj2 "7# years").mp. |  | 22982 |
| 117 | (aged adj2 "8# years").mp. |  | 12386 |
| 118 | (aged adj2 "9# years").mp. |  | 4117 |
| 119 | (elder* adj1 patient?).mp. |  | 60874 |
| 120 | (old adj age).mp. |  | 25856 |
| 121 | (old* adj1 patient?).mp. |  | 72387 |
| 122 | (older adult* or older client* or older patient* or older person* or older people).mp. |  | 123630 |
| 123 | centenarian*.mp. |  | 1742 |
| 124 | elder?.mp. |  | 15623 |
| 125 | elderly.mp. |  | 226662 |
| 126 | geriatri*.mp. |  | 97023 |
| 127 | grandfather*.mp. |  | 1274 |
| 128 | grandma??.mp. |  | 124 |
| 129 | grandmother*.mp. |  | 2421 |
| 130 | grandpa??.mp. |  | 38 |
| 131 | grandparent*.mp. |  | 2918 |
| 132 | nonagenarian*.mp. |  | 1213 |
| 133 | octagenarian*.mp. |  | 38 |
| 134 | oncogeriatri*.mp. |  | 139 |
| 135 | onco-geriatri*.mp. |  | 25 |
| 136 | orthogeriatri*.mp. |  | 313 |
| 137 | ortho-geriatri*.mp. |  | 26 |
| 138 | psychogeriatri*.mp. |  | 1653 |
| 139 | psycho-geriatri*.mp. |  | 119 |
| 140 | retiree*.mp. |  | 1400 |
| 141 | retirement?.mp. |  | 17113 |
| 142 | senior citizen*.mp. |  | 1339 |
| 143 | septuagenarian*.mp. |  | 325 |
| 144 | sexagenarian*.mp. |  | 76 |
| 145 | supercentenarian*.mp. |  | 90 |
| 146 | super-centenarian*.mp. |  | 6 |
| 147 | or/93-146 **[ Elderly ]** |  | 3423860 |
| **148** | **92 and 147 [ Cognition + Questions + Patients/Alternates + Elderly ]** |  | **5756** |
|  |  |  |  |
|  | ***Limits applied:*** |  |  |
| 149 | limit 148 to english language |  | 5239 |
| 150 | exp animals/ not (exp animals/ and exp humans/) |  | 4727456 |
| 151 | 149 not 150 |  | 5236 |
| 152 | limit 149 to humans |  | 5233 |
| 153 | 151 or 152 |  | 5236 |
| 154 | limit 153 to yr="2000 -Current" |  | 4554 |
|  |  |  |  |
|  | ***Update Period:*** |  |  |
| 155 | 20200410:20231231.(da). |  | 312315 |
| 156 | 20200410:20231231.(dt). |  | 46898 |
| 157 | 20200410:20231231.(ed). |  | 331616 |
| 158 | 20200410:20231231.(ep). |  | 26556 |
| 159 | or/155-158 [ Update Period ] |  | 334734 |
| 160 | 154 and 159 [ Base Results + Update Period ] |  | 176 |
| 161 | remove duplicates from 160 🡺 Update Medline Search Results 🡺 |  | 176 |

OVID Search Interface Syntax Guide

| **OVID Search Interface Syntax Guide** | |
| --- | --- |
| Interface: | Ovid |
| Databases: | Embase  MEDLINE  MEDLINE ePubs, In-Process & Other Non-Indexed Citations  Cochrane Database of Systematic Reviews (CDSR)  Cochrane Central Register of Controlled Trials (CCTR)  Ovid EmCare Nursing  PsycINFO (APA)  **Note:** Subject headings have been customized for each database. |
| **SYNTAX GUIDE** | |
| / | At the end of a phrase, searches the phrase or term as a subject heading |
| exp | Explode a subject heading |
| * | Before a word, indicates that the marked subject heading is a primary topic;  or, after a word, a truncation symbol (wildcard) to retrieve plurals or varying endings |
| adj | Requires words are adjacent to each other (in any order) |
| .ti | Title |
| .ab | Abstract |
| .hw | Heading word; usually includes subject headings and controlled vocabulary |
| .pt | Publication type |
| .kw | Author keyword (Embase); Keyword (CDSR an) |
| .kf | Author keyword heading word (MEDLINE) |
| .mp | Mapped term |
| .yr | Year |
| .jw | Journal title word |
