# Supplementary material for: Screening for subjective cognitive decline in the elderly via subjective cognitive complaints and informant-reported questionnaires: a systematic review
Source: BMC Anesthesiol. 2021 Nov 10;21:277. doi: 10.1186/s12871-021-01493-5 (PMC8579566; doi:10.1186/s12871-021-01493-5)
Supplement: Supplementary file 2 — Additional file 2: Table S-2: List of Subjective Cognitive Complaint Questions. [file 12871_2021_1493_MOESM2_ESM.docx]

**“Screening for Subjective Cognitive Decline in the Elderly via Subjective Cognitive Complaints and Informant-Reported Questionnaires: A Systematic Review”**

**Table S2: List of Subjective Cognitive Complaint Questions**

| Study (author, year) | Subjective Cognitive Complaint Questions |
| --- | --- |
| Subjective Cognitive Questions | |
| Bosnes, 2020 | ***MMQ:** This is a test of subjective memory, which contains nine items for assessing memory problems. Each item is scored out of 3 (1 = no problems, 2 = sometimes problems, 3 = often problems), and a total score is produced in the range of 9-27.  1. Memory problems  2. Changed memory  3. Events from minutes ago  4. Remembering names  5. Remembering dates  6. Planned activities  7. Events from days ago  8. Events from years ago  9. Keeping track |
| Hess, 2020 | **SMA:** To assess subjective memory perception, participants completed a 2-item questionnaire. For reach item, they rated level of concern on a 4-point Likert-type scale with scores ranging from 0 to 3.  1. “How would you rate your memory overall?”  2. “How would you rate your memory compared with 10 years ago?” |
| Guerdoux-Ninot, 2019 | **PRMQ:** 16-item measure of prospective and retrospective memory slips in everyday life; 8 address prospective memory failures and 8 address retrospective memory failures:  1. Do they decide to do something in a few minutes time and then forget to do it?  2. Do they fail to recognize a place they have visited before?  3. Do they fail to do something they were supposed to do a few minutes later even though it is there in front of them, like take a pill or turn off the kettle?  4. Do they forget something they were told a few minutes before?  5. Do they forget appointments if they are not prompted by someone else or by a reminder such as a calendar or diary?  6. Do they fail to recognize a character in a radio or television show from scene to scene?  7. Do they forget to buy something they planned to buy, like a birthday card, even when they see the shop?  8. Do they fail to recall things that have happened to them in the last few days?  9. Do they repeat the same story to the same person on different occasions?  10. Do they intend to take something with them, before leaving a room or going out, but minutes later leave it behind, even though it is there in front of them?  11. Do they mislay something that they have just put down, like a magazine or glasses?  12. Do they fail to mention or give something to a visitor that they were asked to pass on?  13. Do they look at something without realizing they have seen it moments before?  14. If they tried to contact a friend or relative who was out, would they forget to try again later?  15. Do they forget what they watched on television the previous day?  16. Do they forget to tell someone something they had meant to mention a few minutes ago? |
| Howland, 2017 | **PROMIS Applied Cognition:** 16 items inquiring about the past 7 days with the following options: never, rarely (once), sometimes (two or three times), often (about once a day), very often (several times a day)  Abilities Subset  1. My mind has been as sharp as usual  2. My memory has been as good as usual  3. My thinking has been as fast as usual  4. I have been able to keep track of what I am doing, even if I am interrupted  5. I have been able to concentrate  6. I have been able to think clearly without extra effort  7. I have been able to pay attention and keep track of what I am doing without extra effort  8. I have been able to remember things as easily as usual without extra effort  Concerns Subset  1. I have had trouble forming thoughts  2. My thinking has been slow  3. My thinking has been foggy  4. I have had trouble concentrating  5. I have had to work really hard to pay attention or I would make a mistake  6. It has seemed like my brain was not working as well as usual  7. I have had to work harder than usual to keep track of what I was doing  8. I have had trouble shifting back and forth between different activities that require thinking |
| Papaliagkas, 2017 | **CFQ**: measure everyday slips of actions and memory failures such as:  Being absent-minded, finding it difficult to concentrate, being forgetful about using things or appointments, not noticing what’s around them, being clumsy, having difficulty making up their mind, being disorganized, getting angry about minor matters  **PRMQ:** 16-item measure of prospective and retrospective memory slips in everyday life; 8 address prospective memory failures and 8 address retrospective memory failures:  1. Do they decide to do something in a few minutes time and then forget to do it?  2. Do they fail to recognize a place they have visited before? 3. Do they fail to do something they were supposed to do a few minutes later even though it is there in front of them, like take a pill or turn off the kettle?  4. Do they forget something they were told a few minutes before?  5. Do they forget appointments if they are not prompted by someone else or by a reminder such as a calendar or diary?  6. Do they fail to recognize a character in a radio or television show from scene to scene?  7. Do they forget to buy something they planned to buy, like a birthday card, even when they see the shop?  8. Do they fail to recall things that have happened to them in the last few days?  9. Do they repeat the same story to the same person on different occasions?  10. Do they intend to take something with them, before leaving a room or going out, but minutes later leave it behind, even though it is there in front of them?  11. Do they mislay something that they have just put down, like a magazine or glasses?  12. Do they fail to mention or give something to a visitor that they were asked to pass on?  13. Do they look at something without realizing they have seen it moments before?  14. If they tried to contact a friend or relative who was out, would they forget to try again later?  15. Do they forget what they watched on television the previous day?  16. Do they forget to tell someone something they had meant to mention a few minutes ago? |
| Markova, 2017 | **QPC:** 2 items inquire about general memory abilities, while the remaining 8 items inquire about particular cognitive complaints with a focus on memory.  The items cover difficulties with: spatial orientation, instrumental activities, personality change, impression of memory change, impression of worse memory in comparison to peers, difficulties with recalling past events, forgetting about appointments, losing things, forgetting about past experiences, limitation in daily activities, words finding difficulties |
| Avila-Villanueva, 2016 | **EMQ:** 28 items for which subjects report how frequently they have memory lapses on a 9-point scale, ranging from "not at all in the last 3 months" to "more than once a day". Lower scores denote better subjective ratings of memory.  Forgetfulness of Immediate Information (FII): 11 items = 2,6,8,13,15,16,17,20,21,23,28  Executive Function (EF): 6 items = 3,4,5,9,10,12  Prospective Memory (PM): 4 items = 7,14,18,22  Forgetfulness of Common Objects (FCO): 2 items = 1, 24  Spatial Orientation (SO): 2 items = 25,26  Note: Items 11,19,27 were excluded from analysis due to skewed values |
| Ramlall, 2013 | **SMC:** single-domain screening measure assessing memory through a “yes”/”no” response to the question:  “Are you experiencing any difficulty with your memory?”  **SMCC:**   1. Difficulty remembering things that had happened in the last few days? 2. Difficulty remembering the names of common objects? 3. Difficulty remembering where you left your belongings? 4. Difficulty remembering the names of people you have known for a long time? 5. Difficulty remembering the names of people who you had met within the last week? 6. Difficulty finding your way around your home? 7. Difficulty finding your way around other places (e.g. shopping center/home of a friend/relative/church)?   **SMRS*** |
| Snitz, 2012 | **SCC:** 24 items had the common prompt and response choices: *“Do you think you are the same, better or worse than you used to be at X?”*.  Item content was weighted toward memory-related changes (e.g., remembering less well than 1 year ago), but also included non-memory items, such as those relating to changes in day-to-day executive functioning and judgment and language  1. Remembering names of people you only met recently?  2. Do you feel you remember things less well than you did a year ago?  3. Finding the right word to use to describe something you know well?  4. Remembering names of people you've known for a long time?  5. Remembering things that happened or were said a few days ago?  6. Remembering a few things you wanted to buy in the store, without writing them down?  7. Remembering where you've put things that you use often?  8. Are you worried about problems with remembering?  9. Solving problems as easily as before?  10. Remembering appointments, messages, etc.?  11. Remembering what day/date/month it is?  12. Remembering when events happened in relation to each other?  13. Remembering telephone numbers of people whom you call often?  14. Remembering the details of a major event?  15. Remembering a familiar/favorite recipe without looking it up?  16. Was this enough of a problem that you talked to your doctor/nurse about it?  17. Keeping up with hobbies and interests?  18. Handling a household emergency like a plumbing leak or a kitchen fire?  19. Remembering a recent major event? (e.g., trip, wedding)  20. Remembering to do important routine things like turn off the stove or lock the door?  21. Remembering things (events, people, etc.) from a long time ago?  22. Remembering how to use familiar appliances, tools, gadgets?  23. Understanding what is going on or being said around you?  24. Getting along with people, or talking and  behaving the way you used to do? |
| Amariglio, 2011 | **SMC:**  1. Have you recently experienced any change in your ability to remember things? [CHANGE IN MEMORY]  2. Do you have more trouble than usual remembering a short list of items, such as a shopping list? [SHORT LIST]  3. Do you have trouble remembering things from one second to the next? [ONE SECOND TO THE NEXT]  4. Do you have much more trouble than usual remembering recent events? [RECENT EVENTS]  5. Do you have any difficulty in understanding or following spoken instructions? [UNDERSTANDING INSTRUCTIONS]  6. Do you have more trouble than usual following a group conversation or plot in a TV program due to your memory? [FOLLOWING A CONVERSATION]  7. Do you have trouble finding your way around familiar streets? [GETTING LOST] |
| Calabria, 2010 | **EMQ:**  *28 items for which subjects report how frequently they have memory lapses on a 9-point scale, ranging from "not at all in the last 3 months" to "more than once a day". Lower scores denote better subjective ratings of memory.*  Forgetfulness of Immediate Information (FII): 11 items = 2,6,8,13,15,16,17,20,21,23,28  Executive Function (EF): 6 items = 3,4,5,9,10,12  Prospective Memory (PM): 4 items = 7,14,18,22  Forgetfulness of Common Objects (FCO): 2 items = 1, 24  Spatial Orientation (SO): 2 items = 25,26  Note: Items 11,19,27 were excluded from analysis due to skewed values |
| Youn, 2009 | **SMCQ**: self-reporting questionnaire for elderly people to assess subjective reports of general memory and everyday memory. 14 items; total score: 14 points. (answers with “yes”/”no” and score of 6 = subjective cognitive decline)  [Global memory functions assessment]  1. Do you think that you have a memory problem?  2. Do you think that your memory is worse than 10 years ago?  3. Do you think that your memory is poorer than that of other people of a similar age?  4. Do you feel that your everyday life is difficult due to memory decline?  [Everyday memory function assessment]  5. Do you have difficulty in remembering a recent event?  6. Do you have difficulty in remembering a conversation from a few days ago?  7. Do you have difficulty in remembering an appointment made a few days ago?  8. Do you have difficulty in recognizing familiar people?  9. Do you have difficulty in remembering where you placed objects?  10. Do you lose objects more often than you did previously?  11. Have you become lost near your home?  12. Do you have difficulty in remembering 2 or 3 items to buy when shopping?  13. Do you have difficulty in remembering to turn off the gas or lights?  14. Do you have difficulty in remembering the phone numbers of your own children? |
| Snitz et al. 2008 | **SMA**  [General questions]  1. In general, how good do you feel your memory is for a person your age?  2. In general, do you feel you remember things less well than you did a year ago? Response choices were “worse,” “same,” or “better” than 1 year ago.  [Specific questions]  1. Remembering things (events, people, etc.) from a long time ago?  2. Remembering things that happened or were said a few days ago?  3. Remembering appointments, messages, etc.?  4. Remembering names of people you’ve known for a long time?  5. Remembering names of people you only met recently?  6. Remembering telephone numbers of people whom you call often?  7. Remembering where you’ve put things that you use often? (keys, watches, etc.)  8. Remembering how to use familiar appliances, tools, gadgets? (includes car)  9. Learning to use new appliances?  10. Finding the right word to use to describe something you know well? (names of familiar objects etc., not names of people)  11. Remembering what day/date it is?  12. Remembering your way around a familiar neighborhood or street?  13. Remembering a few things you wanted to buy in the store, without writing them down?  14. Remember to do important routine things like turn off the stove or lock the door? |
| Lam, 2005 | **A-MIC:** The questions included whether the subjects felt that they were “forgetting where things were placed”, “unable to recall the names of good friends”, “unable to follow and recall conversation”, “having subjective memory problems”, and would “consider own memory to be worse than others of a similar age” |
| Subjective Cognitive Questions and Informant-Reported Questions | |
| Kim, 2019 | **KDSQ-C** - assess memory impairment, language impairment, ability to perform complex tasks in daily life. Has options of “no”/”sometimes”/”often” which are scored as 0,1,2 respectively (score of 6 = dementia)  **AD8** - assess memory changes, orientation, problem-solving ability  1. Problems with judgment (e.g., problems making decisions, bad financial decisions, problems with thinking)  2. Less interest in hobbies/activities  3. Repeats the same things over and over (questions, stories, or statements)  4. Trouble learning how to use a tool, appliance, or gadget (e.g., VCR, computer, microwave, remote control)  5. Forgets correct month or year  6. Trouble handling complicated financial affairs (e.g., balancing check book, income taxes, paying bills)  7. Trouble remembering appointments  8. Daily problems with thinking and/or memory  **SMCQ**** |
| Yim, 2017 | **SMCQ****  **SIRQD**: an informant questionnaire; 15 items; total score: 30 points [NA] |
| Valech, 2015 | **SCD-Q:** Questions assess perceived SCD over the last 2 years, with items addressing changes in everyday memory, language, and executive functions tasks |
| Gavett, 2011 | **IQCODE:**  *Assessing memory and cognitive functions rated from 1 (much better) to 5 (much worse) over last 10 years:*  1. Remembering things about family and friends e.g. occupations, birthdays, addresses  2. Remembering things that have happened recently  3. Recalling conversations a few days later  4. Remembering his/her address and telephone number  5. Remembering what day and month it is  6. Remembering where things are usually kept  7. Remembering where to find things which have been put in a different place from usual  8. Knowing how to work familiar machines around the house  9. Learning to use a new gadget or machine around the house  10. Learning new things in general  11. Following a story in a book or on TV  12. Making decisions on everyday matters  13. Handling money for shopping  14. Handling financial matters e.g. the pension, dealing with the bank  15. Handling other everyday arithmetic problems e.g. knowing how much food to buy, knowing how long between visits from family or friends  16. Using his/her intelligence to understand what is going on and to reason things through |
| Informant-Reported Questions | |
| Tew, 2015 | **AD8:**  1. Problems with judgment (e.g., problems making decisions, bad financial decisions, problems with thinking)  2. Less interest in hobbies/activities  3. Repeats the same things over and over (questions, stories, or statements)  4. Trouble learning how to use a tool, appliance, or gadget (e.g., VCR, computer, microwave, remote control)  5. Forgets correct month or year  6. Trouble handling complicated financial affairs (e.g., balancing check book, income taxes, paying bills)  7. Trouble remembering appointments  8. Daily problems with thinking and/or memory |
| Li, 2013 | **General Practitioner Assessment of Cognition (GPCOG_informant_):**  1. Does the patient have more trouble remembering things that have happened recently?  2. Does he or she have more trouble recalling conversations a few days later?  3. When speaking, does the patient have more difficulty in finding the right word or tend to use the wrong words more often?  4. Is the patient less able to manage money and financial affairs (eg, paying bills, budgeting)?  5. Is the patient less able to manage his or her medication independently?  6. Does the patient need more assistance with transport (either private or public)? |
| Abbate, 2011 | **Interview on Cognitive Status:**  1. Can he/she remember what happened a short time ago? [Anterograde memory]  2. Can he/she remember what happened in the past? [Retrograde memory]  3. Can he/she pay attention and concentrate? [Attention]  4. Has it ever occurred that he/she has made mistakes in performing a specific task or household chores? [Executive functions]  5. Is he/she aware of the cognitive problems you are reporting today? [Awareness]  6. Have you noticed speech problems? [Language]  7. Does he /she get days and dates right? [Temporal orientation]  8. Has he/she retained his/her sense of direction when going out in the neighbourhood? [Spatial orientation] |
| Ayalon, 2011 | 1. “How would you rate your friend’s or relative’s memory at the present time?” Response option is on a 5-point scale, ranging from 1 (excellent) to 5 (poor), with a higher score indicating worse perceived memory  2. Asks informant to compare current functioning to 2 years ago.  3. 16-item questionnaire, 7 measuring memory and 9 measuring IADLs  *Change is rated on a 5-point scale, ranging from 1 (much better) to 5 (much worse). A composite mean score is calculated, with a higher score representing worse perceived cognitive functioning*  1. Remembering things about family and friends e.g. occupations, birthdays, addresses  2. Remembering things that have happened recently  3. Recalling conversations a few days later  4. Remembering his/her address and telephone number  5. Remembering what day and month it is  6. Remembering where things are usually kept  7. Remembering where to find things which have been put in a different place from usual  8. Knowing how to work familiar machines around the house  9. Learning to use a new gadget or machine around the house  10. Learning new things in general  11. Following a story in a book or on TV  12. Making decisions on everyday matters  13. Handling money for shopping  14. Handling financial matters e.g. the pension, dealing with the bank  15. Handling other everyday arithmetic problems e.g. knowing how much food to buy, knowing how long between visits from family or friends  16. Using his/her intelligence to understand what is going on and to reason things through |

**Abbreviations**: AD8, Alzheimer Disease 8; A-MIC, abbreviated memory inventory for the Chinese; CFQ, Cognitive Failures Questionnaire; EMQ, Everyday Memory Questionnaire; GPCOG_informant_, General Practitioner Assessment of Cognition; IADLs - Instrumental Activities of Daily Living; IQCODE, Informant Questionnaire on Cognitive Decline in the Elderly; KDSQ-C, Korean Dementia Screening Questionnaire-Cognition; NR, Not Reported; PRMQ, Prospective and Retrospective Memory Questionnaire; PROMIS, Patient-Reported Outcomes Measurement Information System; QPC, Czech-translated version of Le Questionnaire de Plainte Cognitive; SCC, subjective cognitive complaint; SCD, subjective cognitive decline; SIRQD, Seoul Informant Report Questionnaire for Dementia; SMA, Subjective Memory Assessment; SMC, Subjective Memory Complaint; SMRS, Subjective Memory Rating Scale; SMCC, Subjective Memory Complaint Clinical; SMCQ, Subjective Memory Complaints Questionnaire; MMQ, Meta-Memory Questionnaire

* These questionnaires were not provided.

** These question items are listed under Youn, 2009.
