# Supplementary material for: Screening for subjective cognitive decline in the elderly via subjective cognitive complaints and informant-reported questionnaires: a systematic review
Source: BMC Anesthesiol. 2021 Nov 10;21:277. doi: 10.1186/s12871-021-01493-5 (PMC8579566; doi:10.1186/s12871-021-01493-5)
Supplement: Supplementary file 3 — Additional file 3: Table S-3: Characteristics of Subjective Cognitive Complaint Questionnaire. Abbreviations: AD8: Alzheimer Disease 8; A-MIC, abbreviated memory inventory for the Chinese; CFQ, Cognitive Failures Questionnaire; IADLs - Instrumental Activities of Daily Living; IQCODE, Informant Questionnaire on Cognitive Decline in the Elderly; KDSQ-C, Korean Dementia Screening Questionnaire-Cognition; NR, Not Reported; PRMQ, Prospective and Retrospective Memory Questionnaire; PROMIS, Patient-Reported Outcomes Measurement Information System; SCC, subjective cognitive complaint; subjective cognitive decline, subjective cognitive decline; SIRQD, Seoul Informant Report Questionnaire for Dementia; SMA, subjective memory assessment; SMC, subjective memory complaint; SMCQ, Subjective Memory Complaints Questionnaire; MMQ, Meta-Memory Questionnaire. [file 12871_2021_1493_MOESM3_ESM.docx]

**“Screening for Subjective Cognitive Decline in the Elderly via Subjective Cognitive Complaints and Informant-Reported Questionnaires: A Systematic Review”**

**S-Table 3. Characteristics of Subjective Cognitive Complaint Questionnaire**

| Study (author, year) | Assessment Tool (# of questions) | | Cognitive Measures Assessment Tool | Questions with Positive Association to Cognitive Measures | Prevalence of Complaints (%) | Overall Validation (Yes/No) |
| --- | --- | --- | --- | --- | --- | --- |
|  | | Subjective Cognitive Complaint Questions | | | | |
| Bosnes, 2020 | Meta-Memory Questionnaire (MMQ) (9) | | WMS-III, WAIS-III, and their subsets | NR | NR | Yes |
| Hess, 2020 | Subjective Memory Assessment (2) | | Brief computerized cognitive test; MoCA | NR | NR | NR |
| Guerdoux-Ninot, 2019 | Prospective and Retrospective Memory Questionnaire (PRMQ) (16) | | Neuropsychological battery of tests | NR | NR | Yes |
| Howland, 2017 | PROMIS Applied Cognitive Items (16) | | MMSE; SLUMS | NR | NR | No |
| Markova, 2017 | Czech-translated version of Le Questionnaire de Plainte Cognitive (QPC) (10) | | Neuropsychological battery of tests | Spatial orientation difficulties  Difficulties with recalling past events  Impression of worse memory in comparison to peers with lower memory performance  Limitation in daily activities | Word finding difficulties (40)  Difficulties with recalling past events (32)  Impression of memory change (29)  Losing things (7)  Spatial orientation difficulties (4) | Yes |
| Papaliagkas, 2017 | Cognitive Failures Questionnaire (CFQ) (25)  Prospective and Retrospective Memory Questionnaire (PRMQ) (16)* | | **Neuropsychological battery of tests | NR | NR | Yes |
| Avila-Villanueva, 2016 | Everyday Memory Questionnaire (28)  Everyday Memory Questionnaire (10) | | Neuropsychological battery of tests | Forgetfulness of immediate information (FII)  Executive functions (EF)  Prospective memory (PM) | NR | No |
| Ramlall, 2013 | Subjective Memory Complaint (SMC) (1)*  Subjective Memory Complaint Clinical (SMCC) (7)  Subjective Memory Rating Scale (SMRS) (5)* | | Neuropsychological battery of tests | SMCC:  Difficulty remembering what happened in the last few days  Difficulty remembering the names of people you have known a long time | NR | Yes |
| Snitz, 2012 | Subjective Cognitive Complaint (SCC) (24) | | Neuropsychological battery of tests | Highest α: handling a household emergency  Lowest α: remembering things from a long time ago | Remembering names of people you met only recently (33.4)  Remembering things better now compared to a year ago (30.2)  Worse at getting along with people, talking or behaving the way you used to (1.0) | No |
| Amariglio, 2011 | Subjective Memory Complaint (SMC) (7) | | Neuropsychological battery of tests | Change in memory  Recent events  Understanding events  Following a conversation  Getting lost | Change in memory (56.4)  Remembering items on a short list (29.2)  Forgetting things from one second to the next (25.4)  Recent events (18.5)  Understanding instructions (8.6)  Following a conversation (5.8)  Getting lost (1.6) | No |
| Calabria, 2010 | Everyday Memory Questionnaire (28)  Everyday Memory Questionnaire (20) | | Neuropsychological battery of tests | Memory and learning factor  Learning factor  Language factor  Procedure and monitoring factor  Space factor | NR | No |
| Youn, 2009 | Subjective Memory Complaints Questionnaire (SMCQ) (14) | | SIRQD for cross-validation; standardized Korean version of the CERAD (CERAD-K-N) | Highest correlation: perception of a memory problem, remembering a recent event  Lowest correlation: getting lost near home | NR | Yes |
| Snitz, 2008 | Subjective Memory Assessment (16) | | Neuropsychological battery of tests | Highest association: remembering things that happened or were said a few days ago  Lowest association: finding the right word to describe something | Finding the right word to use to describe something you know well (36.6) | Yes |
| Lam, 2005 | Abbreviated Memory Inventory for the Chinese (MIC) (5) | | Neuropsychological battery of tests | NR | MCIND, MCIID, AD  Subjective memory problems (80.3, 88, 87.3), forgetting where things are placed (68.2, 73.3, 73.2), inability to follow and recall information (48.5, 58, 67.6), inability to recall the names of good friends (39.4, 41.3, 45.1), considering memory to be worse than others of a similar age (30.3, 28, 43.7) | Yes |
|  | | Subjective Cognitive Complaint Questions and Informant-Reported Questions | | | | |
| Kim, 2019 | Korean Dementia Screening Questionnaire-Cognition (KDSQ-C) (15)  Alzheimer Disease 8 (AD8) (8)  Subjective Memory Complaints Questionnaire (SMCQ) (14) | | Neuropsychological battery of tests; clinical diagnosis of dementia (DSM-IV-TR) | NR | NR | Yes |
| Yim, 2017 | Subjective Memory Complaints Questionnaire (SMCQ) - SMC (14)  Seoul Informant Report Questionnaire for Dementia (SIRQD) – Informant-reported (15) | | CERAD-K neuropsychological battery | NR | NR | Yes |
| Valech, 2015 | Subjective Cognitive Decline Questionnaire (SCD-Q), “MyCog” – SMC (24)  Subjective Cognitive Decline Questionnaire (SCD-Q), “TheirCog” – informant-reported (24) | | Neuropsychological battery of tests and psychological assessment; AD biomarkers | NR | NR | Yes |
| Gavett, 2011 | Informant Questionnaire on Cognitive Decline in the Elderly (IQCODE) (16) | | Neuropsychological battery of tests | NR | NR | No |
|  | | Informant-Reported Questions | | | | |
| Tew, 2015 | Alzheimer Disease 8 (AD8) (8) | | MMSE; Recall tests; Copy tests | NR | NR | Yes |
| Li, 2013 | General Practitioner Assessment of Cognition (GPCOG_informant_) (6) | | MMSE; HDS; DSM-IV for validation | NR | NR | Yes |
| Abbate, 2011 | Interview on Cognitive Status (8) | | Telephone interview | Anterograde memory  Retrograde memory  Attention  Awareness  Temporal orientation  Spatial orientation | Anterograde memory (91.6)  Attention deficits (63.5)  Executive function (46.2)  Retrograde memory (30.3),  Awareness (28.6)  Language/spatial orientation/spatial abilities (21.8)  Temporal orientation (11.8) | Yes |
| Ayalon, 2011 | 1 Single-Item Informant Measure (1)* | | Neuropsychological battery of tests | NR | NR | Yes |

**Abbreviations:** PROMIS, Patient-Reported Outcomes Measurement Information System; WAIS, Wechsler adult intelligence scale; WMS, Wechsler Memory Scale; MCIIND, mild cognitive impairment possible incipient dementia; MCIND, mild cognitive impairment not demented; MCIID, MCI possible incipient dementia; MMSE, mini-mental state examination; SLUMS, Saint Louis University Mental Status; DSM, diagnostic and statistical manual of mental disorders; HDS, Hasegawa dementia scale, MoCA, Montreal cognitive assessment; NR, Not Reported.

*Questionnaire included subjective memory complaint items only.

**Tests included in the neuropsychological test batteries differ depending on the study, but they commonly included free and cured memory recall, verbal and attention tests.
